# Spatiotemporal Assessment and Driving Factors of Ecosystem Health: A Case Study of Two Provinces in Southern China

**DOI:** 10.3390/biology14060671

**Published:** 2025-06-09

**Authors:** Yujun Cai, Yu Zhang, Yefeng Jiang, Xi Guo

**Affiliations:** 1 Technology Innovation Center for Land Spatial Ecological Protection and Restoration in Great Lakes Basin, MNR, Jiangxi Agricultural University, Nanchang 330045, China; caiyujun809@gmail.com (Y.C.); guoxi@jxau.edu.cn (X.G.); 2 Technology Innovation Center for Land Spatial Ecological Protection and Restoration in Great Lakes Basin, MNR, Nanchang 330029, China; 3 Key Laboratory of Poyang Lake Watershed Agricultural Resources and Ecology (Co-Construction by Ministry and Province), Ministry of Agriculture and Rural Affairs, Jiangxi Agricultural University, Nanchang 330045, China

**Keywords:** ecosystem health, VORS framework, random forest model, shapley additive explanations, driving factors

## Abstract

Ecosystem health (EH) is critically linked to regional sustainable development, ecological security, and human well-being. However, studies on spatiotemporal assessment and driving factors of EH at the provincial level are lacking. Taking Jiangxi and Hunan provinces in China as the study areas, we proposed a framework for the spatiotemporal assessment and driving factors of EH by incorporating the Vigour–Organisation–Resilience–Services framework, the Random Forest model, and Shapley Additive explanations. Our results indicated that ecosystem resilience, organisation, vigour, and services in the study area exhibited an overall upward trend from 2000 to 2020. Notably, these indicators experienced declines during certain periods, and these declines coincided with the rapid urbanisation phases in China, highlighting the significant pressure of urbanisation on EH. Furthermore, EH in the study area showed a fluctuating upward trend from 2000 to 2020, with the mean EH value increasing from 0.721 to 0.738. The spatial distribution pattern was characterised by “low values in the central region and high values in the surrounding areas”. Specifically, high-value areas were primarily concentrated in the regions with a high density of ecological land, such as forests, while low-value areas closely corresponded to the regions with a high density of built-up land. In addition, the terrain ruggedness index, potential crop yield, and population density were the three most influential factors affecting EH in the study area from 2000 to 2020. Notably, key drivers exhibited nonlinear interactions, particularly between natural and socioeconomic factors, indicating that EH dynamics were shaped more by their combined effects than by individual variables alone. Overall, the proposed framework can be used for spatialtemporal assessment of EH, while clarifying the driving mechanism of EH. The time, space, model, and environmental variables involved can be freely replaced in similar regions.

## Simple Summary

Previous studies on ecosystem health often faced limitations in terms of spatiotemporal scales, driving factors, or combinations of these aspects. Here, we aimed to construct a comprehensive framework for spatiotemporal assessment and driving factors of ecosystem health, with Jiangxi and Hunan provinces as the study areas. Our results found that ecosystem health in the study area showed a downward trend during the period of rapid urban growth. Overall, areas with more forests tend to be healthier, while areas with more buildings tend to be less healthy. The most important factors affecting ecosystem health are terrain, agricultural productivity, and population levels, which often interact with each other and exhibit nonlinear characteristics. Our study provides a comprehensive framework for assessing the health status of ecosystems and their driving factors across spatiotemporal scales, which can help governments and planners balance economic development and ecological protection and make better decisions.

## 1. Introduction

The concept of ecosystem health (EH), proposed in the 1980s [1], emphasises the integrity, functionality, and resilience of ecosystems, forming a conceptual foundation for environmental management [2,3]. As ecosystem degradation accelerates, the Global Assessment Report by the Intergovernmental Science-Policy Platform on Biodiversity and Ecosystem Services [4] emphasises the urgent need to incorporate EH evaluation into policy to safeguard ecosystem services. There has been growing international interest in incorporating EH into sustainability policy and practice. For instance, Charron [5] advanced the integration of EH into global sustainability agendas through participatory and interdisciplinary ecosystem-based frameworks, while Lu et al. [6] highlighted the need to identify key ecological drivers, emphasising sustainability and resilience as essential links between natural systems and human well-being. These global efforts have underscored the need for a clearer conceptual understanding of EH and a practical evaluation framework. EH refers to an ecosystem’s ability to maintain its internal structure and functions, as well as its capacity to adapt to external changes and disturbances [7,8]. Healthy ecosystems typically exhibit stable biodiversity, normal ecological processes, moderate resource utilisation, and adaptability to environmental changes [9,10]. Spatially, EH is highly variable, as it is influenced by both natural and socioeconomic factors within specific regional landscapes [11,12,13]. Investigating EH is critical for improving regional ecosystem service capacity and harmonising environmental sustainability with socioeconomic development. Therefore, establishing a framework for the spatiotemporal assessment and driving factors of EH has become a key research focus [14,15].

Evaluation frameworks for EH have evolved in distinct stages, with continuous refinements and innovations introduced by researchers. In the 1970s, researchers [16,17] introduced the pressure–state–response framework and its extensions to analyse the mechanisms of human impacts on ecosystems. These frameworks emphasise how external pressures drive changes in ecosystem states and prompt societal responses. However, these frameworks primarily emphasise external disturbances while overlooking intrinsic ecosystem health and natural processes. By the 1990s, several researchers [15,18,19] had proposed the Vigour–Organisation–Resilience (VOR) framework to provide a more holistic assessment of EH. This framework measures biological vigour, structural integrity, and resilience and provides a more ecologically oriented evaluation approach. Although the VOR framework has been widely applied in practice, it does not fully account for the impact of human activities on the assessment process. In the 21st century, as research has progressed, many researchers [20,21] have developed a comprehensive evaluation system based on natural, social, and economic subsystems that take regional sustainable development as its core and provide a more holistic assessment of EH. Notably, this evaluation system shares significant similarities with the sustainable development assessment framework, making it difficult to capture accurately the essential characteristics of regional EH [22,23]. In summary, different EH evaluation frameworks have their unique characteristics but also exhibit certain limitations. To address these gaps, recent studies [24,25] proposed the Vigour–Organisation–Resilience–Services (VORS) framework, which extends the VOR framework by incorporating the relationship between ecosystem service provision and human needs, thereby expanding the socioeconomic dimension of EH assessment. Although the VORS framework addresses some of the limitations of the VOR framework, it focuses primarily on health status evaluation and does not fully reveal the driving mechanisms underlying EH changes.

In recent years, various methods have been developed to explore the mechanisms driving the changes in EH. Traditional models such as Geographically and Temporally Weighted Regression (GTWR) have been used to assess spatial heterogeneity [16,26,27], while Geographical Convergent Cross Mapping (GCCM) has improved causal inference under limited samples [28]. However, both approaches face limitations in handling high-dimensional, nonlinear interactions. As data availability continues to increase, an increasing number of researchers have turned to machine learning methods to identify driving factors. Compared to traditional models, machine learning methods such as Random Forest and XGBoost can handle larger datasets and offer a more comprehensive quantification of driving factor influences. Moreover, machine learning models can be combined with SHapley Additive exPlanations (SHAP) to further quantify the marginal contribution of each variable and improve interpretability. For instance, Zhang et al. [29] integrated XGBoost with SHAP to identify the key drivers of EH changes and visualise variable interactions. However, their approach focused primarily on variable importance and lacked a more systematic explanation of the underlying mechanisms. Xu et al. [30] further improved this by combining Random Forest with SHAP in the context of ecosystem services, producing more interpretable and reliable insights into driver contributions and interactions. However, this method has not yet been applied to the integrated analysis of EH and its complex driving mechanisms, especially at the provincial level.

To fill this gap, taking Jiangxi and Hunan provinces in China as the study areas, we proposed a framework for the spatiotemporal assessment and driving factors of EH. Jiangxi and Hunan, as representative provinces where economic development and environmental protection are closely linked in China, offer valuable cases for investigating the driving mechanisms of EH [18]. Specifically, our objectives are as follows: (1) to analyse the spatiotemporal evolution of EH from 2000 to 2020 in the Jiangxi and Hunan provinces; (2) to identify key drivers of EH change using an integrated Random Forest and SHAP approach; and (3) to ultimately develop a comprehensive and transferable evaluation framework for EH, applicable to regions balancing economic development and environmental protection. This study not only contributes to methodological advancement in EH assessment but also supports more effective ecological governance in one of China’s most dynamic ecological–economic transition zones.

## 2. Materials and Methods

### 2.1. Study Area

**Figure 1:**
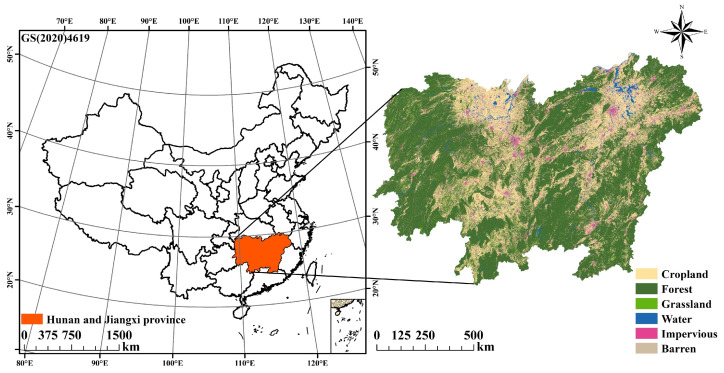
Location and land use types of the study area.

The study area encompasses Jiangxi and Hunan provinces (Figure 1), which are located in the middle and lower reaches of the Yangtze River in China. The topography in the study area is complex and diverse, including mountains, hills, basins, plains, and water bodies. The region experiences a humid subtropical monsoon climate, with hot, wet summers and mild winters; annual average temperatures range from 16 °C to 25 °C, and precipitation falls between 1200 mm and 1700 mm. Notably, the region faces increasing interannual climate variability and seasonally concentrated rainfall, which heighten the sensitivity of ecosystems to both natural and anthropogenic stressors [31].

Land use in this area is undergoing rapid transformation, with extensive urban expansion and infrastructure development leading to substantial reductions in cropland and natural land cover [32]. These land-use transitions, coupled with socioeconomic development pressures, have resulted in pronounced trade-offs in ecosystem services (ESs), including declines in habitat quality and water purification functions, especially in urban agglomeration areas [18,33]. Notably, Xu’s research shows that the urbanisation changes in Hunan and Jiangxi provinces have the most prominent impact on EH in the Yangtze River Economic Belt [30]. However, spatiotemporal assessment and driving factors of EH in the study area are lacking. Therefore, conducting EH assessment and analysis in the study area is crucial for ecological restoration and economic development.

### 2.2. Study Framework

**Figure 2:**
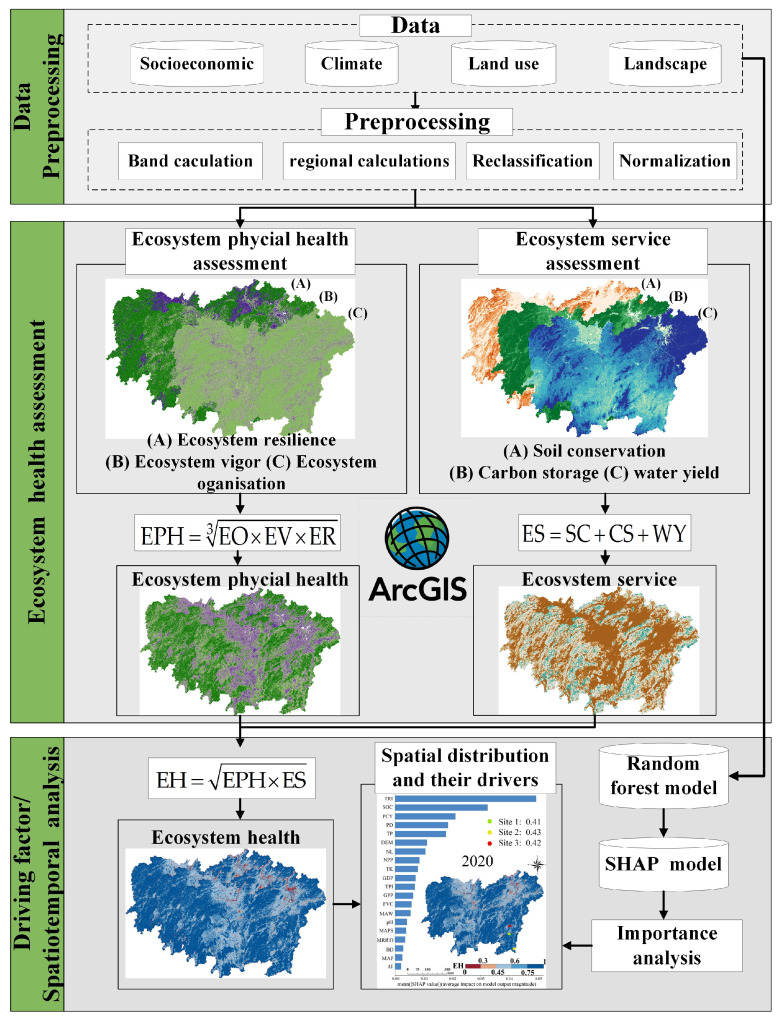
Technical roadmap for spatiotemporal assessment and driving factors of ecosystem health.

The framework of this study is illustrated in Figure 2. First, we collected multi-source data, including land use, climate, landscape, and socioeconomic factors. These data underwent preprocessing steps, such as band calculations, regional calculations, reclassification, and normalisation, to derive key indicators. Those indicators include ecosystem resilience (ER), ecosystem organisation (EO), ecosystem vigour (EV), soil conservation, carbon storage, and water yield. Next, we calculated ecosystem physical health (EPH) using ER, EO, and EV, and ecosystem services (ESs) were assessed based on soil conservation, carbon storage, and water yield. By integrating these two components, we constructed a comprehensive evaluation system to assess the EH in the study area. Building on this, we analysed the spatiotemporal variations in key indicators and EH levels to reveal long-term trends and spatial distribution patterns. Lastly, we applied the Random Forest model to identify the key driving factors influencing EH and used SHAP to quantify the contribution and interaction of each factor, thereby uncovering the driving mechanisms behind EH changes. Our framework not only integrates EV, EO, ER, and ES for a comprehensive assessment but also leverages machine learning models to enhance the accuracy of driving factor identification. The results of this study provide a scientific basis for ecological protection and management.

### 2.3. Data Source and Processing

The data used in our study mainly included the EH evaluation and driving factor data. The sources of EH evaluation data are as follows: (1) Normalised Difference Vegetation Index data and carbon density data of various land cover types were obtained from the National Ecological Science Data Centre (https://www.nesdc.org.cn, on 22 December 2024); (2) mean annual precipitation, digital elevation model, and rainfall erosivity factor data were obtained from the National Earth System Science Data Centre (https://www.geodata.cn, on 23 December 2024); (3) mean annual potential evapotranspiration data were obtained from the National Tibetan Plateau/Third Pole Environment Data Centre (https://data.tpdc.ac.cn, on 23 December 2024); and (4) other data, such as land use data, soil data, maximum root depth, plant available water content, root depth, vegetation transpiration coefficient, soil and water conservation measures factor, and vegetation cover factor, were derived from relevant literature, FAO crop reference values, and InVEST model user guides [34,35,36,37,38]. Additionally, the soil erosion factor was calculated based on soil texture and referenced from previous studies [39]. The sources of driving factors data are as follows: (1) mean annual pressure, mean annual wind speed, mean annual temperature, annual minimum temperature, and nightlight data were obtained from the National Tibetan Plateau/Third Pole Environment Data Centre (https://data.tpdc.ac.cn, on 23 December 2024); (2) aspect, slope, slope length, profile curvature, plan curvature, general curvature, digital elevation model, multiresolution index of ridge top flatness, terrain ruggedness index, and multiresolution index of valley bottom flatness data were obtained from the National Earth System Science Data Centre (https://www.geodata.cn, on 23 December 2024); (3) net ecosystem productivity, net primary productivity, gross primary productivity, mean annual aridity index, mean annual precipitation, potential crop yield, and tree cover data we obtained from the Resource and Environment Science Data Platform (http://www.resdc.cn, on 23 December 2024); (4) population density data were obtained from the LandScan database (https://landscan.ornl.gov, on 23 December 2024); (5) fractional vegetation cover data were obtained from the National Ecological Science Data Centre (https://www.nesdc.org.cn, on 22 December 2024); and (6) soil data (soil total phosphorus, soil pH, soil total nitrogen, cation exchange capacity, soil total potassium, and soil bulk density) and GDP data were derived from previous studies [37,38,40]. The specific data types and sources are listed in Table 1 and Table 2, respectively.

### 2.4. Assessment of Ecosystem Health

#### 2.4.1. Assessment of Ecosystem Physical Health

EPH was evaluated through the integration of EV, EO, and ER [41] using the following equation:

(1) $$\mathrm{EPH}=\sqrt[{3}]{\mathrm{EO}\times \mathrm{EV}\times \mathrm{ER}}$$

where EPH is the ecosystem physical health level, EO is the ecosystem organisation level, EV is the ecosystem vigour level, and ER is the ecosystem resilience level.

EO reflects the structural stability of a regional ecosystem, including landscape heterogeneity and connectivity. Spatial-adjacency-based pattern indices have been widely applied to measure EO. Landscape heterogeneity was characterised by the Shannon evenness index and the area-weighted mean patch fractal dimension. Landscape connectivity was quantified using the contagion and fragmentation indices [41]. The weighting scheme for the four selected indicators was determined through expert consultation and by referring to previous studies [42,43] conducted in the Yangtze River Basin and other subtropical monsoon regions to ensure that each metric’s contribution to landscape structural stability was appropriately reflected. All indices were calculated based on the land use/land cover data (Table 1), using FRAGSTATS version 4.2—a widely adopted software for spatial pattern analysis of categorical raster maps. Before aggregation, all four landscape indices were normalised using the min–max normalisation method to eliminate the effects of differing value ranges and ensure comparability across indicators. The specific calculation formula is as follows:

(2) EO=0.3SHDI +0.2AWMPFD +0.25FN +0.25CONT 

where SHEI is the Shannon evenness index, AWMPFD is the area-weighted mean patch fractal dimension, CONT is the contagion index, and FN is the fragmentation index.

EV represents the primary productivity and metabolic capacity of an ecosystem. Our study adopted the NDVI to quantify EV [44]. The NDVI is a robust indicator of vegetation growth status and coverage, directly reflecting the efficiency of light-to-chemical energy conversion and the availability of ecosystem resources for human use. NDVI has been widely used as an indicator of vegetation growth due to its accessibility, high temporal resolution, and reliable performance in subtropical monsoon regions such as the Yangtze River Basin. In these areas, consistent seasonal vegetation patterns and dense canopy coverage enhance its reliability as an indicator [45,46,47]. While NPP provides a direct measure of plant productivity, its coarse spatial resolution and limited data availability at the regional scale make it less suitable for our study. Thus, NDVI serves as a more practical alternative.

ER describes the ability of an ecosystem to resist disturbances and recover its structure and function. Therefore, they can be divided into the capacity to resist external disturbances and the ability to recover their original state after the ecosystem is damaged. We used resistance to represent the former and resilience to describe the latter. Land-use types were assigned weights for resistance and recovery based on methods adopted from previous studies [48,49,50] on ecosystem health and spatial resilience, as summarised in Table S1. These coefficients, although fixed for each land-use category, are empirically derived and have been widely applied in similar regional assessments. The ER index was calculated as a weighted composite of recovery and resistance, with a 0.6:0.4 ratio based on previous studies [44] that applied similar schemes in the context of ecosystem resilience evaluation.

ER was calculated as follows:

(3) $$\mathrm{ER}=0.6\times \sum P_{i}\times Resil_{i}+0.4\times \sum P_{i}\times Resist_{i}$$

where $P_{i}$ represents the ${\;⠀}_{i}$-th pixel, and ${\mathrm{Resil}}_{i}$ and ${\mathrm{Resist}}_{i}$ are the resilience coefficient and resistance coefficient of the ${\;⠀}_{i}$-th land use type, respectively.

#### 2.4.2. Assessment of Ecosystem Services 

ES was evaluated based on three critical functions, carbon storage, water yield, and soil conservation, using spatial overlay analysis to integrate spatial distribution patterns and assess regional ES levels. Since the precise contribution weights of these three dominant services could not be determined, we did not assign specific weights to each function. Instead, to ensure objectivity and reliability, the overall ES was calculated as the arithmetic mean of the three services. This approach has been widely applied in prior studies when weighting schemes are uncertain, as it helps reduce subjectivity and maintain comparability across services [51,52].

In ES research, the carbon storage function is typically quantified using the carbon storage and sequestration module in the InVEST model. The total carbon stock in the ecosystem is calculated using carbon density data for different land use types. The calculation of carbon stock is as follows:

(4) $$C_{\mathrm{total}}=\sum_{i=1}^{n}A_{i}\cdot \left(C_{\mathrm{above},i}+C_{\mathrm{below},i}+C_{\mathrm{soil},i}+C_{\mathrm{dead},i}\right)$$

where $A_{i}$ is the area (ha) of the *i* land use type; $C_{\mathrm{above},i}$, $C_{\mathrm{below},i}$, $C_{\mathrm{soil},i}$, and $C_{\mathrm{dead},i}$ represent the carbon densities of aboveground biomass, belowground biomass, soil carbon, and litter carbon, respectively (Mg C/ha). Carbon density values were obtained from the National Ecological Science Data Centre of China (Table 1). Annual land use data from 2000 to 2020 were used to estimate interannual variation in carbon storage. The carbon density of each land use category is assumed to remain constant across all years, and changes in total carbon stock were therefore attributed solely to land use transitions. As this study focuses on spatiotemporal variation rather than absolute carbon stock values, no regional calibration or uncertainty analysis was conducted. This approach is consistent with previous studies [53,54] using the InVEST model, which prioritises relative change over precise quantification.

The soil conservation function refers to the ability of surface vegetation, such as forests and grasslands to reduce soil erosion and effectively prevent nutrient loss. It is an essential regulatory element of the ES. Current studies commonly use the SDR module of the InVEST model to calculate soil conservation based on a universal soil loss equation. The calculation involved the interception of sediments and particles by upstream grids to determine the potential and actual soil erosion. The difference between the two indicated the extent of soil conservation.

(5) $$\begin{array}{l} RKLS=R\times K\times LS\\ USLE=R\times K\times LS\times C\times P\\ SD=RKLS-USLE \end{array}$$

where RKLS represents the potential soil erosion, R is the rainfall erosivity factor [MJ·mm/(ha·h)], obtained from datasets provided by the National Tibetan Plateau Data Centre (Table 1), K is the soil erodibility factor [t·ha·h/(ha·MJ·mm)], calculated using the erosion–productivity impact model proposed by Williams, based on soil texture, organic carbon content, and bulk density data (Table 1), LS is the slope length and steepness factor, extracted from the digital elevation model using the built-in terrain analysis function of the InVEST SDR module, USLE is the actual soil erosion amount, and P and C are the conservation practices and vegetation cover factors, respectively. These were assigned based on expert consultation and previous studies [35,36], considering the relative effectiveness of soil and water conservation practices across different land use types in the study area (Table S2).

The water yield function refers to an ecosystem’s ability to retain water over a specific period under natural conditions, which is essential for providing water for human production and daily life. This function is influenced by multiple factors such as hydrology, climate, vegetation, and soil. The water yield module in the InVEST model is based on the Budyko water–energy balance model, utilising parameters such as precipitation, maximum soil root depth, plant-available water, and potential evapotranspiration to calculate the water yield. The formula used was as follows:

(6) $$\begin{array}{l} Y(x)=\left(1-\frac{AET(x)}{P(x)}\right)\times P(x)\\ \frac{AET(x)}{P(x)}=1+\frac{PET(x)}{P(x)}-{\left[1+{\left(\frac{PET(x)}{P(x)}\right)}^{w}\right]}^{\frac{1}{w}}\;\\ PET(x)=K_{c}(l_{x})\times ET_{o}(x)\\ w(x)=Z\times \frac{AWC(x)}{P(x)}+1.25 \end{array}$$

where Y(x) represents the annual water yield for the grid cell, AET(x) is the annual evapotranspiration (mm), obtained from the National Tibetan Plateau Data Centre (Table 1), P(x) is the annual precipitation (mm), sourced from the National Earth System Science Data Centre (Table 1), w is a nonphysical parameter representing climate–soil interactions, and $K_{c}(l_{x})$ is the vegetation-specific evapotranspiration coefficient, calculated based on land use type concerning expert consultation (Table S3) and previous study [35]. $ET_{o}(x)$ is the reference crop evapotranspiration coefficient, derived from the FAO crop reference values, and AWC(x) is the plant-available water (mm) estimated using soil texture data following methods from previous study [36]. Z is a seasonal constant with a range of 0 < Z < 30. The value of *Z* was calibrated through iterative testing based on regional hydrological characteristics and previous studies [39] and was set to 16.

#### 2.4.3. Assessment of Ecosystem Health

A healthy ecosystem is defined as one that is active and capable of maintaining its organisational structure, self-regulation, and recovery under external disturbances while sustainably fulfilling reasonable human demands. In other words, it is stable and sustainable. Therefore, we evaluated EPH based on EV, EO, and ER and also assessed ES based on soil conservation, carbon storage, and water yield. In this study, we combined EPH and ES to evaluate EH, and the calculation formula is shown as follows:

(7) $$\mathrm{EH}=\sqrt{\mathrm{EPH}\times \mathrm{ES}}$$

where EPH represents the ecological physical health index derived from the integration of EV, EO, and ER, and ES denotes the value of ecosystem services.

### 2.5. Analysis of Drivers

#### 2.5.1. Random Forest

Random Forest, proposed by Breiman, is an ensemble learning algorithm that constructs multiple uncorrelated decision trees via bootstrap sampling to enhance prediction accuracy and stability [55]. It is robust against overfitting, handles high-dimensional data efficiently, and captures nonlinear relationships. We utilised the Random Forest (RF) model to identify the key drivers of EH dynamics.

To implement the model, we first defined the dependent and independent variables. The dependent variable was the EH value for a specific year, extracted from the EH distribution map. Independent variables are listed in Table 2. The modelling was conducted at the grid cell level; approximately 10% of grid cells were randomly sampled from the raster data, and 1000 samples were obtained after thinning (Figure S1). All predictor variables were normalised, and outliers were removed during preprocessing. The dataset was then randomly split into training and testing sets in an 8:2 ratio. A Random Forest model was trained, and a grid search was used to optimise hyperparameters to improve model performance.

Following model training, we evaluated its predictive performance to ensure the robustness of the results. The coefficient of determination (*R*^2^) was used as the primary measure of model accuracy. The specific formula for calculating *R*^2^ is as follows:

(8) $$R^{2}=1-\frac{\sum_{i=1}^{n}{\left(y_{i}-{\hat{y}}_{i}\right)}^{2}}{\sum_{i=1}^{n}{\left(y_{i}-\bar{y}\right)}^{2}}$$

where $y_{i}$ is the actual observed value (the EH-observed value), ${\hat{y}}_{i}$ is the predicted value (the EH-predicted value), $\bar{y}$ is the mean of the observed values, and *n* is the sample size.

#### 2.5.2. Shapley Additive Explanations

To further quantify the impact of each driving factor on EH changes and explore their interaction mechanisms, our study introduced SHAP based on the RF model. SHAP allocates an output value to each feature using Shapley values to measure the influence of each driver on the final output value [29], as calculated in the following equation:

(9) $$g(z)=\phi_{0}+\sum_{j=1}^{M}\phi_{j}z_{j}$$

where *M* is the number of driving factors, $z_{j}$ is the set of factors excluding, and $\phi_{j}$ is the Shapley value for the *j*-th driver; $\phi_{0}$ is a constant.

In this study, SHAP was implemented using the TreeSHAP algorithm, which is specifically optimised for tree-based models such as Random Forest. For each time point (2000, 2005, 2010, 2015, and 2020), an independent RF model was trained, and SHAP values were calculated based on the same set of driving variables.

## 3. Results

### 3.1. Spatiotemporal Changes in Ecosystem Physical Health

**Figure 3:**
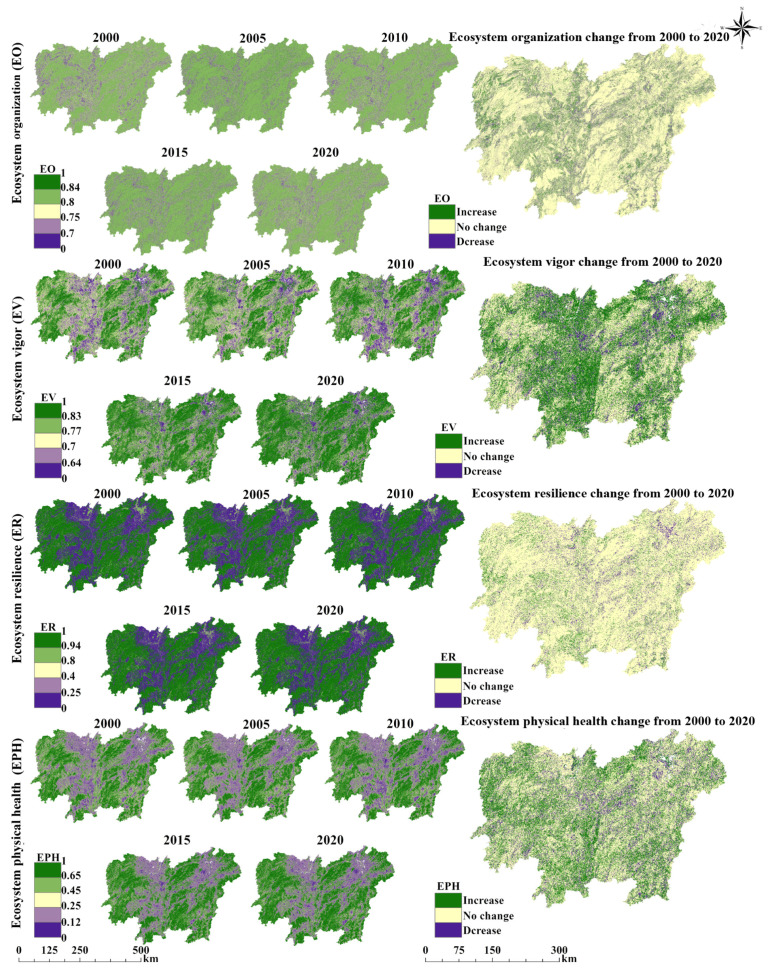
Spatiotemporal changes in ecosystem physical health. EO: ecosystem organisation, EV: ecosystem vigour, indicating the productivity and vitality of ecosystems. ER: ecosystem resilience, EPH: ecosystem physical health. Pixels classified as “no change” were defined by an absolute interannual difference threshold of |ΔX| < 0.005. Considering the 20-year study period, this magnitude of change is assumed to lie within the range of natural variability or model-induced uncertainty and thus does not indicate a meaningful trend.

The spatiotemporal evolution of ecological indicators in the study area from 2000 to 2020 reveals distinct distributional trends and variation patterns. Figure 3 illustrates the spatial distribution patterns of EO, EV, ER, and EPH, which exhibited a “low in the centre, high around the edges” trend from 2000 to 2020 in the study area. Notably, the high-value areas of each indicator largely correspond to forest land distribution, while the low-value areas align with the construction land distribution. The average EO values ranged from 0.7877 to 0.8104 in the study period 2000–2020, indicating a relatively high level. EO increased from 2000 to 2005, followed by a continuous decline over the next 15 years (Table S4). Overall, the EO in the study area improved slightly from 2000 to 2020. A spatial assessment over the past two decades revealed that areas with declining EO were concentrated in the central part of the study area. Meanwhile, EV exhibited a fluctuating upward trend over the 20 years, with its average value increasing from 0.7512 to 0.7859. Specifically, EV rose from 2000 to 2005, declined over the next five years, and then maintained a steady upward trend from 2010 to 2020. During this period, the low-value areas of EV gradually decreased, whereas the high-value areas expanded. Notably, the fluctuating increase in EV is primarily concentrated in the eastern part of the study area. Our findings also indicate that ER remained above 0.72 on average, from 2000 to 2020, reflecting a relatively high level. In terms of trends, ER slightly declined from 2000 to 2005 but then increased continuously over the following 15 years, reaching its highest value of 0.7525 in 2020. Although ER showed an overall upward trend, its spatial distribution pattern remained largely unchanged. The average EPH values for 2000, 2005, 2010, 2015, and 2020 were 0.4516, 0.4599, 0.4591, 0.4814, and 0.4845, respectively, indicating moderate levels (Table S4). In terms of temporal changes, the EPH followed a trend similar to that of EV. Apart from a slight decline between 2005 and 2010, it exhibited an upward trend during the other periods. Overall, EPH exhibited an “increase in the east, decline in the centre” pattern over the past two decades.

### 3.2. Spatiotemporal Changes in Ecosystem Service

**Figure 4:**
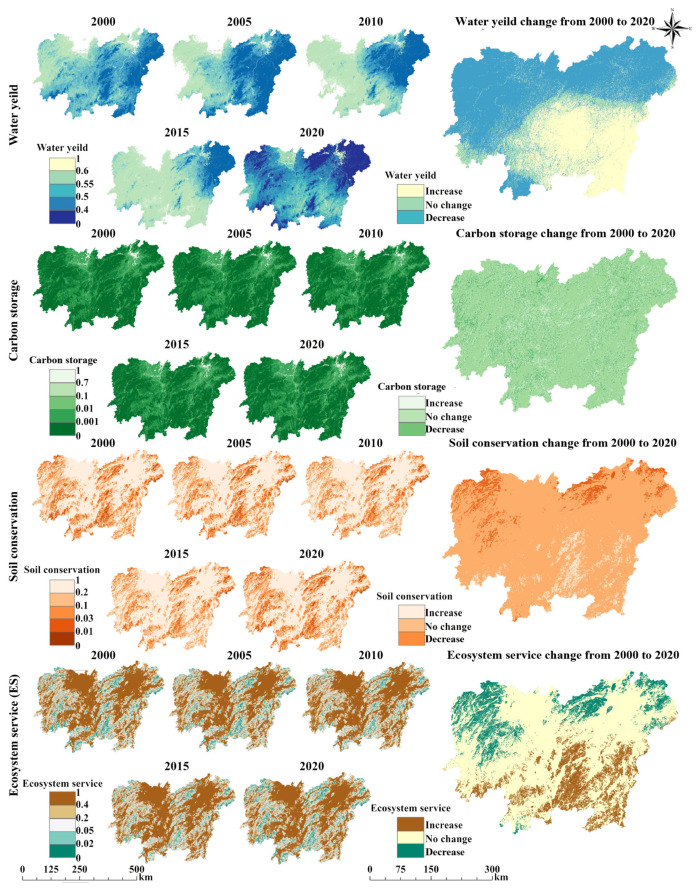
Spatiotemporal changes in ecosystem services. Pixels classified as “no change” were defined by an absolute interannual difference threshold of |ΔX| < 0.01. Considering the 20-year study period, this magnitude of change is assumed to lie within the range of natural variability or model-induced uncertainty and thus does not indicate a meaningful trend.

The changes in water yield, carbon storage, soil conservation, and ES levels in the study area from 2000 to 2020 are shown in Figure 4. Among these, the water yield level exhibits a “low in the west, high in the east” spatial distribution pattern (Figure 4). The average values for each year were 0.5381, 0.5420, 0.5107, 0.4884, and 0.5676, respectively (Table S5). The water yield level continuously declined from 2000 to 2015 but increased significantly from 2015 to 2020, reaching its highest value in the two decades by 2020. Overall, the spatiotemporal assessment indicates a “northward increase, southward decrease” trend. Additionally, both carbon storage and soil conservation levels exhibit a “low in the centre, high around the edges” spatial distribution pattern, similar to EPH (Figure 4). In terms of temporal trends, carbon storage exhibited a continuous decline. Soil conservation followed a similar pattern to that of water yield, decreasing from 2000 to 2015 before increasing significantly from 2015 to 2020, reaching 0.0292 (Table S5). Overall, carbon storage remained relatively stable, with declines primarily concentrated in the central region, whereas soil conservation increased in the north and decreased in the south.

The results in Figure 4 also indicate that the ES changes between 2000 and 2020 share different characteristics with the other three indicators. The spatial distribution of ES aligns with that of carbon storage and soil conservation, displaying a “low in the centre, high around the edges” pattern. Temporally, ES continuously declined from 2000 to 2015 but experienced significant growth from 2015 to 2020 (Table S5), mirroring the trends in water yield and soil conservation. Overall, the ES level in the study area showed a slight improvement from 2000 to 2020, displaying a “northward increase and a southward decrease” trend, similar to soil conservation. However, influenced by fluctuations in water yield and carbon storage, ES levels exhibited greater variation than soil conservation.

### 3.3. Spatial Distribution of Ecosystem Health and Their Driving Factors 

**Figure 5:**
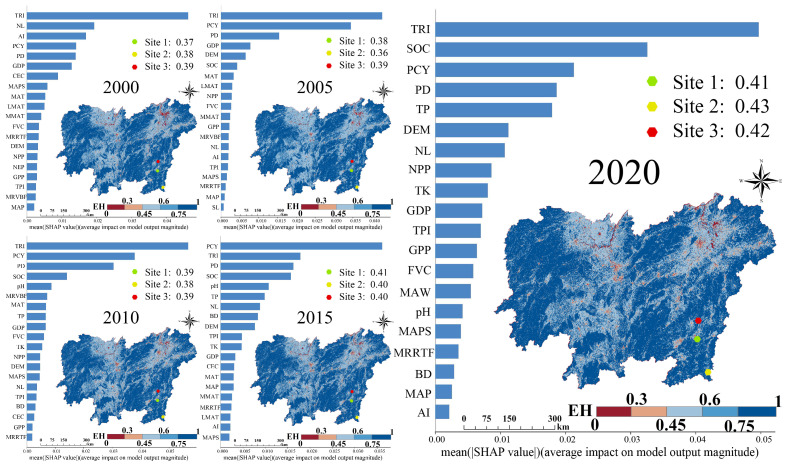
Spatiotemporal distribution and driving factors of ecosystem health. The red, yellow, and green points indicate the locations of three mine ecological restoration verification sites. Bar charts show the top-ranked drivers of EH for the years 2000, 2005, 2010, 2015, and 2020 based on average absolute SHAP values, indicating the relative contribution of each factor to the EH prediction. SHAP values are unitless and reflect the magnitude of feature impact on the model’s output; higher values indicate a stronger influence. The adjacent maps display the spatial distribution of EH for each corresponding year, with colour gradients representing different EH levels. All axes, colour bars, and legends are annotated for clarity.

**Figure 6:**
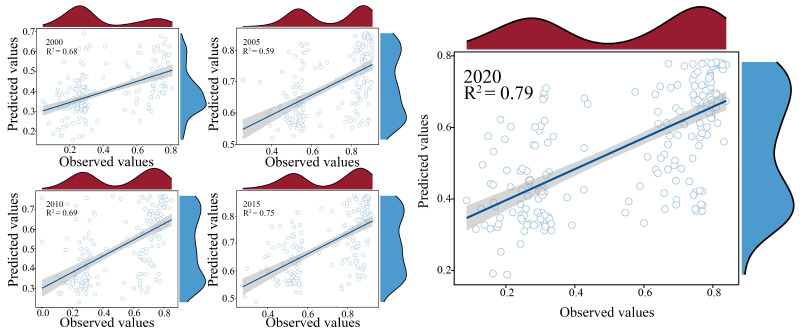
Scatter plot of ecosystem health modelling based on random forest. Each subplot includes a linear regression fit (black line) with a 95% confidence interval (shaded area), along with the coefficient of determination (R^2^) indicating model performance. Marginal density plots along the top and right sides show the distribution of observed and predicted values.

The results in Figure 5 indicate that EH in the study area exhibited a spatial distribution pattern similar to that of EPH, characterised by “low in the centre, high around the edges” from 2000 to 2020. In terms of temporal trends, the average EH in the study area decreased only between 2005 and 2010 but showed an upward trend in all other years. The overall spatiotemporal changes over the 20 years indicate a fluctuating but generally increasing trend in the EH. To qualitatively validate our model outputs, we selected three representative mine ecological restoration areas as verification sites (Figure 5) and conducted field investigations. On-site landscapes of the three sites are shown in Figures S2, S3, and S4, respectively. The EH values in those areas remained consistently within a low range (0.30–0.45) from 2000 to 2020, which aligns with its known history of ecological degradation and gradual recovery. This consistency supports the reliability and spatial rationality of the model results. Meanwhile, we also evaluated the model from the perspective of overall predictive accuracy. The results in Figure 6 show that the goodness of fit (R^2^) of the RF model ranged between 0.59 and 0.79, indicating a good model fit. Therefore, we further applied the SHAP analysis to interpret the RF results and quantify the impact of various driving factors on EH over time.

As shown in Figure 5, TRI, NL, AI, PCY, and PD were the five most important driving factors in 2000. In 2005, the rankings shifted to TRI, PCY, PD, GDP, and DEM. By 2010, the ranking had changed to TRI, PCY, PD, SOC, and pH. In 2015, the top five factors were PCY, TRI, PD, SOC, and pH, whereas in 2020, they were TRI, SOC, PCY, PD, and TP. Notably, TRI, PCY, and PD consistently appeared in the top five rankings across all years, highlighting their importance in influencing EH. The SHAP values for TRI ranged from 0.18 to 0.52. Except for 2015, TRI had the highest impact on EH in most years, indicating its dominant role. The SHAP values for PCY were 0.18, 0.37, 0.38, 0.38, and 0.24. Although its influence was lower than that of TRI, PCY still played a significant role in EH changes, ranking higher than most of the other driving factors. The SHAP values for PD fluctuated around 0.2, showing a consistent influence on EH, although their importance was lower than those of TRI and PCY. Additionally, NL and GDP had significant impacts on EH in 2000 and 2005, but their SHAP values remained low in the subsequent years. In contrast, the effects of SOC, pH, and TP on EH followed the opposite trends. Their influence was relatively low in 2000 and 2005 but increased in later years, as reflected by the rising SHAP values. These results suggest that, among the socioeconomic factors, soil-related variables representing land sociocarrying capacity have become increasingly significant in influencing EH over time. To further explore the influence patterns of the driving factors, we generated SHAP dependence plots and interaction heatmaps across five time points. Using PCY as an example (Figure S5), the dependence plots revealed a nonlinear relationship where the positive effect of PCY on EH weakened with increasing values. At the same time, the interaction effects with other driving factors, such as TRI and NL, have also significantly diminished its positive impact. SHAP interaction heatmaps (Figure S6) further showed that interactions among key variables, especially between PCY, TRI, and PD, intensified from 2000 to 2020. Additionally, the SHAP-derived feature rankings were consistent with Random Forest importance scores (Figure S7), supporting the robustness of our attribution results.

## 4. Discussion

Based on the traditional VORS framework, our study analysed the spatiotemporal assessment of EH in the study area from the perspective of ecosystem complexity by integrating multiple geographic spatial datasets. In addition, the RF model and SHAP were employed to quantify the influence of various driving factors on EH across different years. Our results indicated that both natural ecological and socioeconomic factors significantly affect EH, with socioeconomic factors having the greatest influence. Furthermore, the relative importance of variables representing land social carrying capacity among socioeconomic factors showed an increasing trend from 2000 to 2020, highlighting the growing role of land use change in the variation in EH. Wang et al. [16] used a GTWR model and found that both natural ecological and socioeconomic factors significantly influenced EH, which is consistent with our findings. Guo et al. [15] analysed the driving factors of EH changes using the GCCM and concluded that socioeconomic factors had a more significant impact on EH changes, which is consistent with our study. However, their studies did not fully quantify the degree of influence of each driving factor. In this study, we utilised the RF model combined with SHAP, which not only overcame the limitations of traditional methods in high-dimensional variable analysis but also effectively quantified the degree of influence of each driving factor under the influence of nonlinear relationships between different factors. Compared with the GCCM and GTWR models, which rely on specific assumptions or geographic weight structures, the RF model combined with SHAP avoids information loss caused by dimensionality reduction, providing higher analysis precision and variable resolution capacity, thereby offering a more comprehensive and in-depth understanding of the driving mechanisms of EH changes.

Moreover, our results also found that the EH of the study area remained relatively stable but exhibited a slowly increasing trend from 2000 to 2020. Notably, the EPH, ES, and EH declined between 2005 and 2010. This period coincides with the accelerated urbanisation phase in China, during which rapid land-use changes [56,57,58] led to a decline in EPH and ES, eventually resulting in a drop in EH. Meanwhile, EH, EPH, and ER exhibited a significant synergistic relationship in their spatial distribution: all three showed a “low in the centre, high around the edges” spatial pattern. The low-value areas for the three factors were highly consistent with the spatial distribution patterns of EO, EV, carbon storage, water yield, and ES. This consistency suggests that the spatial distribution of ER has a decisive influence on the spatial distributions of EPH and EH, as indicated by previous study [44]. Furthermore, ER is directly related to land-use types within the region, with the concentration of construction land in the central area leading to lower ER values, which subsequently contributes to the spatial clustering of low EH values. In summary, the results of our study suggest that a high concentration of construction land was the main reason for the low EH values in the study area and that the rapid expansion of construction land exceeded the ecosystem balance threshold during 2005–2010, causing a decline in EH. Similar studies have found that urban expansion is a major threat to the ecosystems of urbanised areas, reducing ES and ultimately damaging EH [59,60,61]. Therefore, balancing the human–environment relationship should become a core decision-making focus. On the one hand, unregulated urban sprawl should be strictly controlled through spatial land planning; on the other hand, the construction and protection of land types with high vegetation cover, such as urban forests and grasslands, should be strengthened. Sustainable development can only be achieved by coordinating socioeconomic development with ecosystem protection.

Our results also suggested that TRI, PCY, and PD are important in EH. Among them, TRI had the greatest impact, followed by PCY, while PD had the least impact. Notably, TRI, as a major natural ecological factor, influences EH by affecting landscape connectivity in the study area, ultimately becoming the key natural factor driving changes in EH. However, among the top five driving factors for EH changes each year, natural ecological factors accounted for only one or two factors, and, except for TRI, the influence of other natural factors on EH was relatively low. Therefore, we conclude that socioeconomic factors play a more significant role than natural ecological factors in changes in EH. This phenomenon may be closely related to the rapid expansion of urban land, which has accelerated changes in the supply and demand balance of ES, making socioeconomic factors more significant in influencing EH. This conclusion is consistent with the research conducted in other regions [62,63]. To further interpret these results, we examined the SHAP dependence and interaction plots. For example, PCY showed a generally linear negative relationship with EH, its actual influence was significantly moderated by nonlinear interactions with terrain such as TRI and human activity indicators such as NL and PD. In areas with high terrain ruggedness or dense human settlement, the positive effect of PCY weakened substantially, reflecting the complexity of how ecological and anthropogenic factors jointly shape EH. These compound effects became more pronounced over time, as also reflected in the SHAP interaction heatmaps. Such patterns emphasise the importance of not only identifying key drivers but also understanding their interactive dynamics within specific spatial and developmental contexts. Similar interactions have been reported in previous studies [30]. Additionally, we found that from 2000 to 2020, with socioeconomic development, the importance of NL and GDP declined, whereas the importance of variables representing the land’s social carrying capacity, such as SOC, pH, and TP, increased. This trend may be due to rapid urbanisation, leading to the unregulated expansion of construction land, where a lack of spatial planning constraints often causes land resource misallocation and inefficient utilisation. In this context, the regional economic development model has gradually shifted from extensive expansion to more refined utilisation [64].

In terms of study limitations, although the RF model and SHAP can accurately predict the relationship between EH changes and their driving factors and reliably predict the significance of each driving factor, these models have limitations in analysing local spatial heterogeneity. As a result, they may not fully capture the complexity and diversity of geographic spatial data, potentially affecting the precision of the local spatial feature analysis. Additionally, our validation was limited to only three field points, which may not be sufficient to comprehensively assess model performance. Therefore, future research should incorporate more validation points and adopt broader and more systematic validation approaches. This includes integrating other spatial analysis models and employing multiple validation techniques, such as cross-validation and sensitivity analysis, to better reveal causal relationships at varying spatial scales, offering new perspectives for exploring the links between EH and its driving factors. 

## 5. Conclusions

In this study, we integrated a comprehensive assessment framework for EV, EO, ER, and ES with multi-source remote sensing data and machine learning methods, such as the RF model and SHAP, systematically revealing the spatiotemporal assessment of EH and its driving mechanisms in the Jiangxi and Hunan provinces of southern China from 2000 to 2020. Our results show the following: (1) ER, EO, EV, and ES in the study area exhibited overall upward trends from 2000 to 2020. Notably, these indicators experienced declines during certain periods, and these declines coincided with the rapid urbanisation phases in China, underscoring urbanisation’s significant pressure on EH. (2) EH in the study area showed a fluctuating upward trend from 2000 to 2020, with a spatial distribution pattern characterised by “low values in the central region and high values in the surrounding areas”. High-value areas were primarily concentrated in regions with a high density of ecological land, such as forests, whereas low-value areas closely corresponded to regions with a high density of built-up land. (3) TRI, PCY, and PD were the three most influential factors affecting EH in the study area from 2000 to 2020. Among these, TRI played the dominant role, followed by PCY, whereas PD had the weakest influence. However, among the top five driving factors, socioeconomic factors dominated, suggesting that socioeconomic factors had a more significant impact on EH than natural ecological factors during the study period. Through multi-scale and multi-factor comprehensive analyses, this study deepens our understanding of the spatiotemporal assessment of EH and its driving mechanisms. These findings not only enrich theoretical research on EH but also provide scientific support for the formulation of differentiated ecological protection and restoration measures.

## Figures and Tables

**Table 1:** Factors used to evaluate ecosystem health in the study area.

Data Name	Period	Resolution	Data Source
Land use/land cover (LULC)	2000–2020	30 m	[34]
Normalised Difference Vegetation Index	2000–2020	30 m	National Ecological Science Data Centre (https://www.nesdc.org.cn, on 22 December 2024)
Carbon density of land cover types	/	1000 m	
Mean annual precipitation	2000–2020	1000 m	National Earth System Science Data Centre (https://www.geodata.cn, on 23 December 2024)
Digital elevation model	/	250 m	
Rainfall erosivity factor	2000–2020	1000 m	
Annual average potential Evapotranspiration	2000–2020	1000 m	National Tibetan Plateau/Third Pole Environment Data Centre (https://data.tpdc.ac.cn, on 23 December 2024)
Soil data (soil texture, soil organic carbon, and soil bulk density)	1980s/2010s	1000 m/250 m	[37,38]
Plant-available water content	/	/	[36]
Root depth	/	/	[35]
Maximum root depth, vegetation transpiration coefficient, soil and water conservation measures factor, and vegetation cover factor	/	/	[35,36]; FAO crop reference values and InVEST model user guide
Soil erosion factor	1980s/2010s	1000 m/250 m	Calculated from soil texture data [39]

**Table 2:** Environmental covariates used to evaluate drivers of ecosystem health in the study area.

Driving Factors	Abbreviation	Time Period	Resolution	Data Source
Mean annual press	MAPS	2000–2020	1000 m	National Tibetan Plateau/Third Pole Environment Data Centre (https://data.tpdc.ac.cn, on 23 December 2024)
Mean annual wind	MAW	2000–2020	1000 m	
Mean annual temperature	MAT	2000–2020	1000 m	
Annual minimum temperature	LMAT	2000–2020	1000 m	
Night light	NL	2000–2020	1000 m	
Aspect	Aspect	/	250 m	National Earth System Science Data Centre (https://www.geodata.cn, on 23 December 2024)
Slope	Slope	/	250 m	
Slope length	SL	/	250 m	
Terrain ruggedness index	TRI	/	30 m	
Profile curvature	PCE	/	250 m	
Plan curvature	PC	/	250 m	
General curvature	GC	/	250 m	
Digital elevation model	DEM	/	250 m	
Multiresolution index of ridge top flatness	MRRTF	/	250 m	
Multiresolution index of valley bottom flatness	MRVBF	/	250 m	
Net primary productivity	NEP	2000–2020	900 m	
Net ecosystem productivity	NPP	2000–2020	900 m	
Gross primary productivity	GPP	2000–2020	900 m	
Aridity index	AI	2000–2020	1000 m	
Mean annual precipitation	MAP	2000–2020	1000 m	
Potential crop yield	PCY	2000–2020	1000 m	Resource and Environment Science Data Platform (http://www.resdc.cn, on 23 December 2024)
Tree cover	TC	2000–2020	1000 m	
Population density	PD	2000–2022	1000 m	(https://landscan.ornl.gov, on 23 December 2024)
Fractional vegetation cover	FVC	2000–2020	250 m	National Ecological Science Data Centre (https://www.nesdc.org.cn, on 22 December 2024)
Soil total phosphorus	TP	1980s/2010s	1000 m/250 m	[37,38]
Soil pH	pH	1980s/2010s	1000 m/250 m	
Soil total nitrogen	TN	1980s/2010s	1000 m/250 m	
Cation exchange capacity	CEC	1980s/2010s	1000 m/250 m	
Soil total potassium	TK	1980s/2010s	1000 m/250 m	
Soil bulk density	BD	1980s/2010s	1000 m/250 m	
Real GDP	GDP	2000–2019	1000 m	[40]

## Data Availability

The data presented in this study are available upon request from the corresponding author.

## Supplementary Materials

The following supporting information can be downloaded at: https://www.mdpi.com/article/10.3390/biology14060671/s1, Figure S1: Spatial distribution of grid-based sampling points used for Random Forest model training and validation; Figure S2: Field survey photograph of Site 1; Figure S3: Field survey photograph of Site 2; Figure S4: Field survey photograph of Site 3; Figure S5: SHAP dependence plots of PCY, SOC, and pH across selected time points. Each plot illustrates the relationship between feature values (PCY, SOC, or pH) and their SHAP impact on EH predictions. The *x*-axis represents the feature value, and the *y*-axis indicates the corresponding SHAP value. Color gradients represent the values of the most relevant interacting variables, enabling the visualization of potential interaction effects at different time points; Figure S6: SHAP interaction heatmap of driving factors across five time points. Each heatmap shows the magnitude of pairwise interaction effects between variables on EH predictions, revealing dynamic and non-linear relationships over time; Figure S7: Top 20 Random Forest Variable Importances across Five Time Points. The ranking of variable importances based on the Random Forest model is shown for five time points. The top 20 variables are presented, and their relative importance scores are listed. The ranking results show a consistent trend with those derived from SHAP values, indicating robust identification of influential features. Table S1: Ecosystem resilience (ER) coefficients of each land use type; Table S2: Vegetation cover (C) and conservation practice (P) factors under different land use types; Table S3: Vegetation-specific evapotranspiration coefficient(Kc) under different land use types; Table S4: Average values of Ecosystem organisation, Ecosystem vigour, Ecosystem resilience, and Ecosystem physical health from 2000 to 2020; Table S5: Average values of water yield, carbon storage, soil conservation, and Ecosystem service from 2000 to 2020.
