# Supplementary material for: Spatiotemporal Assessment and Driving Factors of Ecosystem Health: A Case Study of Two Provinces in Southern China
Source: Biology (Basel). 2025 Jun 9;14(6):671. doi: 10.3390/biology14060671 (PMC12189917; doi:10.3390/biology14060671)
Supplement: Supplementary file 1 [file biology-14-00671-s001.zip › biology-3630503-supplementary.pdf]

## Supplementary Text

**Table S1.** Ecosystem resilience (ER) coefficients of each land use type

| Landscape type | Grassland | Farmland | Forest | Barren land | Impervious land | Water |
|---|---|---|---|---|---|---|
| 0.6×Resil | 0.8 | 0.3 | 0.6 | 0.4 | 0.2 | 0.7 |
| 0.4×Resist | 0.6 | 0.5 | 0.6 | 1 | 0.3 | 0.8 |

**Table S2.** Vegetation cover (C) and conservation practice (P) factors under different land use types

| Landscape type | Grassland | Farmland | Forest | Barren land | Impervious land | Water |
|---|---|---|---|---|---|---|
| C | 0.06 | 0.23 | 0.05 | 1 | 0 | 0 |
| P | 0.35 | 0.75 | 0.15 | 1 | 1 | 1 |

**Table S3.** Vegetation-specific evapotranspiration coefficient(Kc) under different land use types

| Landscape type | Grassland | Farmland | Forest | Barren land | Impervious land | Water |
|---|---|---|---|---|---|---|
| Kc | 0.65 | 0.85 | 0.65 | 1 | 0.25 | 0.2 |

**Table S4.** Average values of Ecosystem organisation, Ecosystem vigour, Ecosystem resilience, and Ecosystem physical health from 2000 to 2020

| Year | Ecosystem organisation | Ecosystem vigour | Ecosystem resilience | Ecosystem physical health |
|---|---|---|---|---|
| 2000 | 0.787743581 | 0.751220381 | 0.740225547 | 0.451683382 |
| 2005 | 0.810374568 | 0.758644127 | 0.726433664 | 0.459940126 |
| 2010 | 0.796936612 | 0.752936262 | 0.74030257 | 0.459078071 |
| 2015 | 0.802804899 | 0.784579602 | 0.74407964 | 0.481387127 |
| 2020 | 0.796242232 | 0.785944838 | 0.752549944 | 0.484581907 |

**Table S5.** Average values of water yield, carbon storage, soil conservation, and Ecosystem service from 2000 to 2020

| Year | Water yield | Carbon storage | Soil conservation | Ecosystem service |
|---|---|---|---|---|
| 2000 | 0.538107647 | 0.850336146 | 0.02901522 | 0.0870456620 |
| 2005 | 0.542035752 | 0.84853945 | 0.027702007 | 0.08310602 |
| 2010 | 0.510713651 | 0.843573303 | 0.022490793 | 0.067472378 |
| 2015 | 0.488433839 | 0.837649983 | 0.021741211 | 0.065223632 |
| 2020 | 0.567619845 | 0.83496228 | 0.029177653 | 0.087532959 |

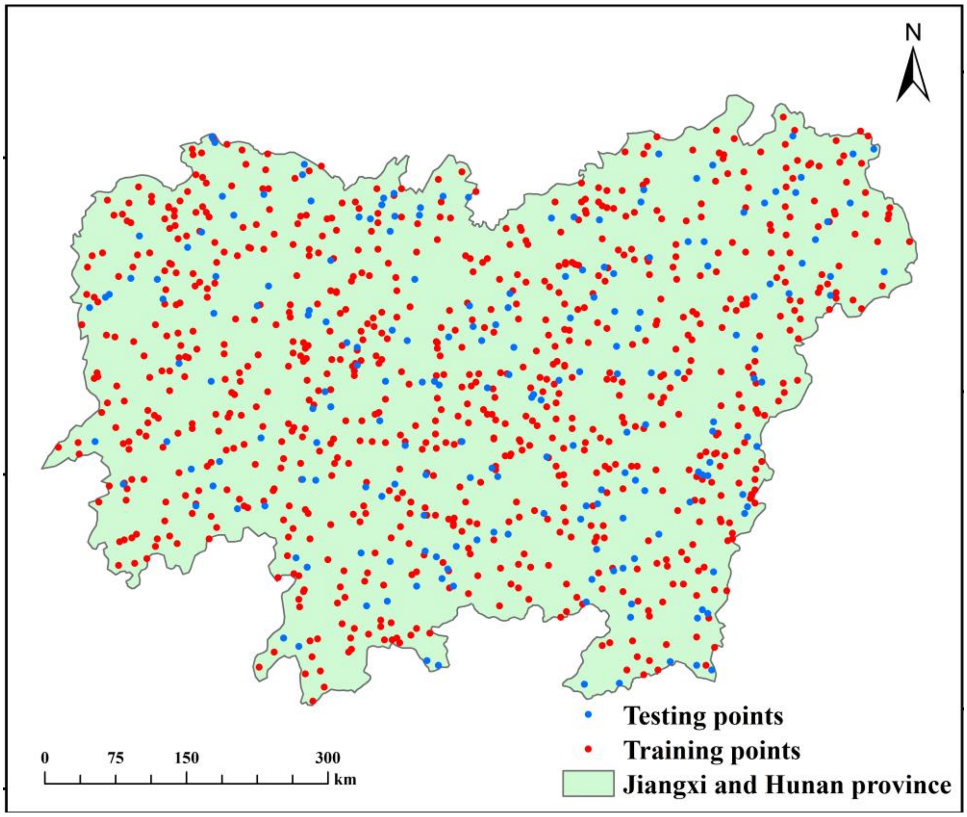


**Figure S1.** Spatial distribution of grid-based sampling points used for Random Forest model training and validation.

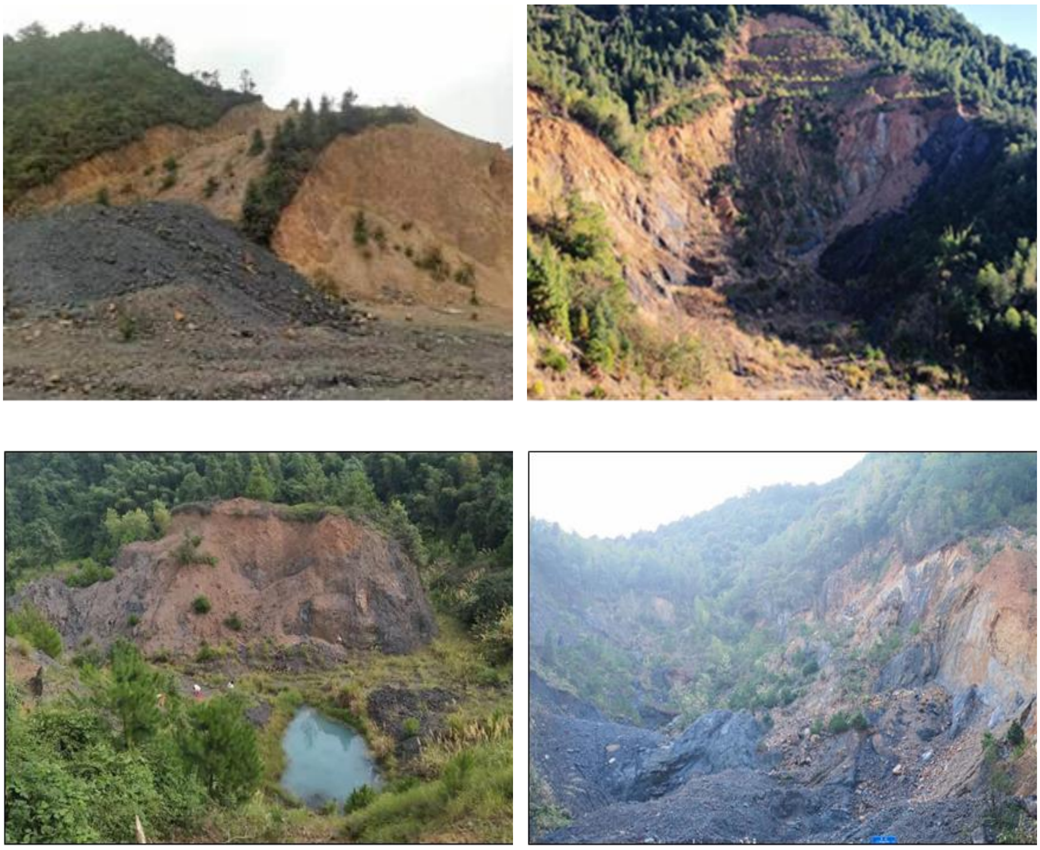

**Figure S2.** Field survey photograph of Site 1

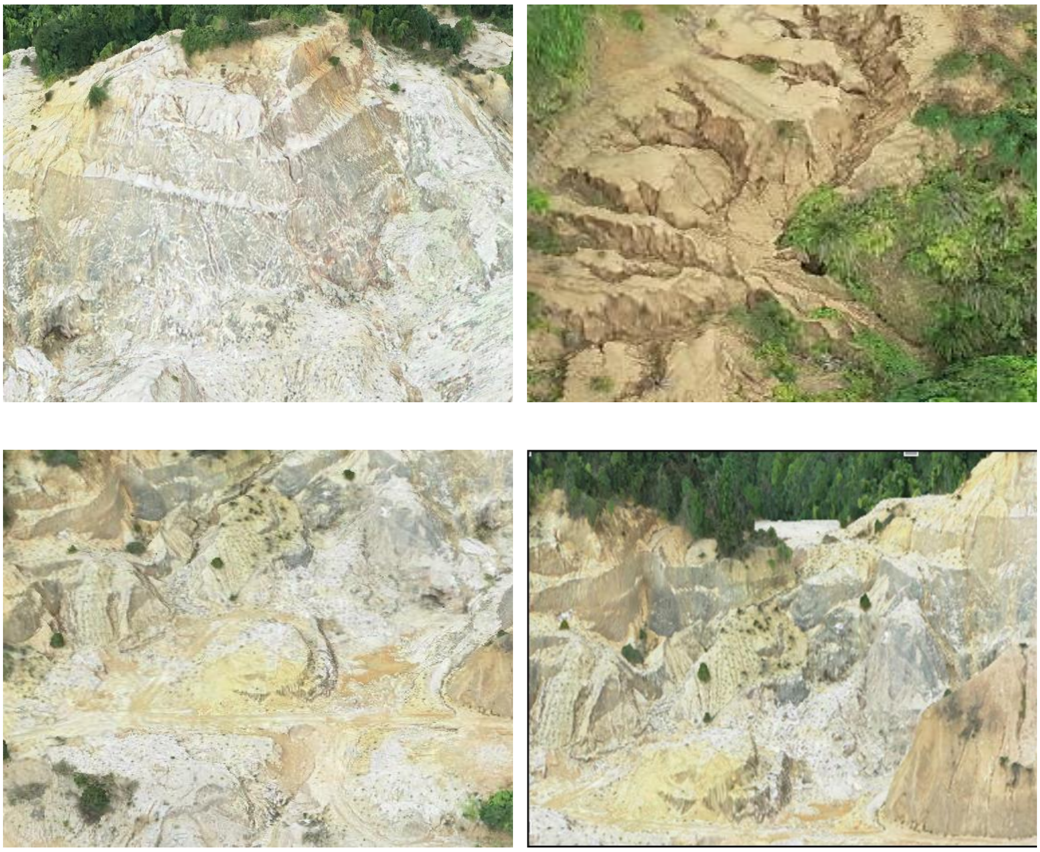

**Figure S3.** Field survey photograph of Site 2

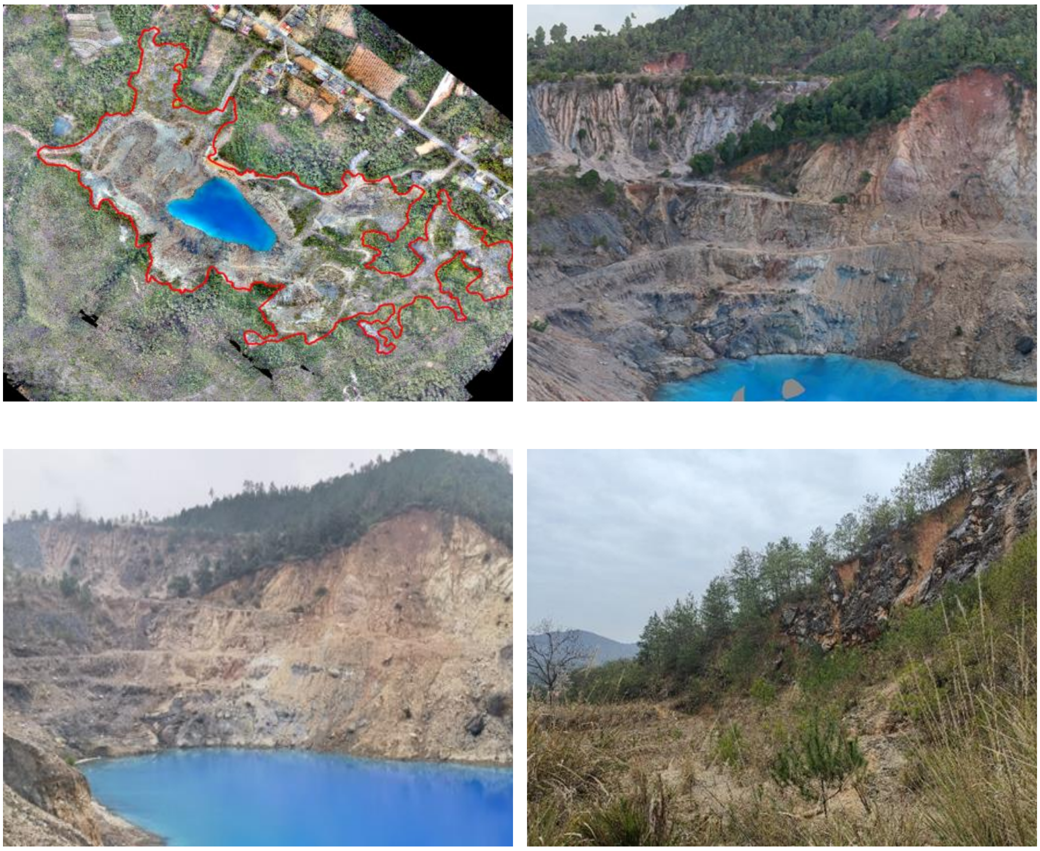

**Figure S4.** Field survey photograph of Site 3

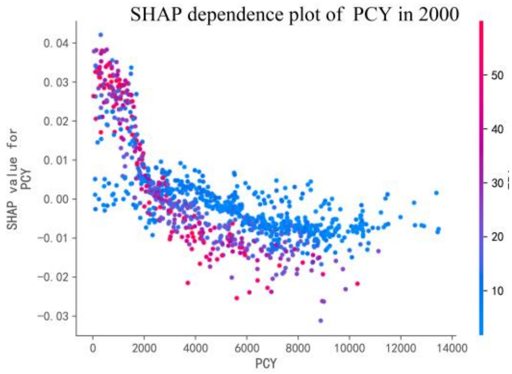
SHAP dependence plot of PCY in 2000
SHAP value for PCY
PCY

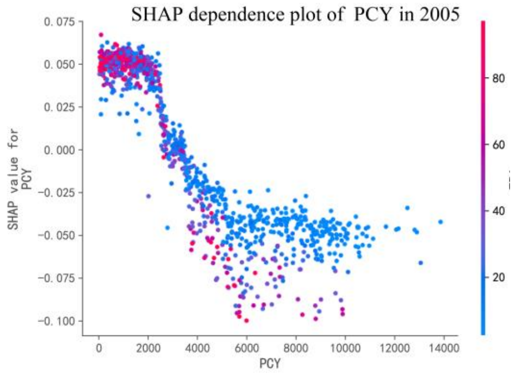
SHAP dependence plot of PCY in 2005
SHAP value for PCY
PCY

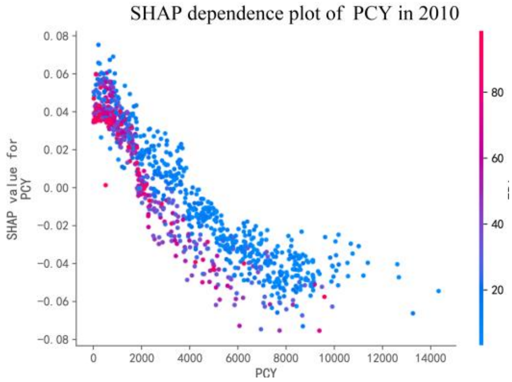
SHAP dependence plot of PCY in 2010
SHAP value for PCY
PCY

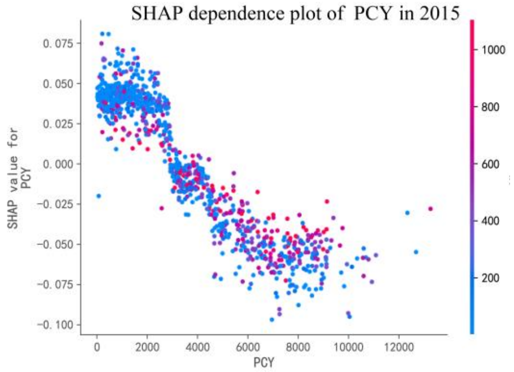
SHAP dependence plot of PCY in 2015
SHAP value for PCY
PCY

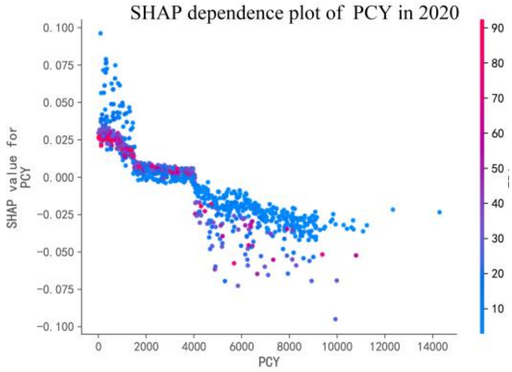
SHAP dependence plot of PCY in 2020
SHAP value for PCY
PCY

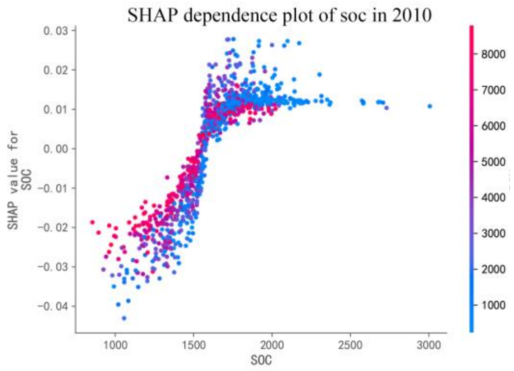
SHAP dependence plot of soc in 2010
SHAP value for SOC
SOC

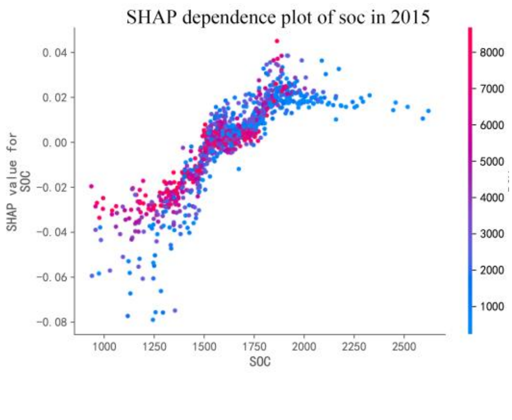
SHAP dependence plot of soc in 2015
SHAP value for SOC
SOC

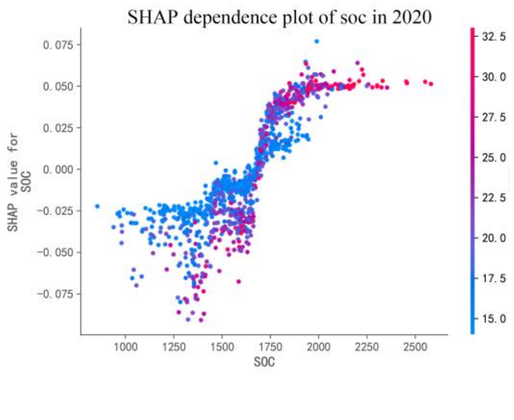
SHAP dependence plot of soc in 2020
SHAP value for SOC
SOC

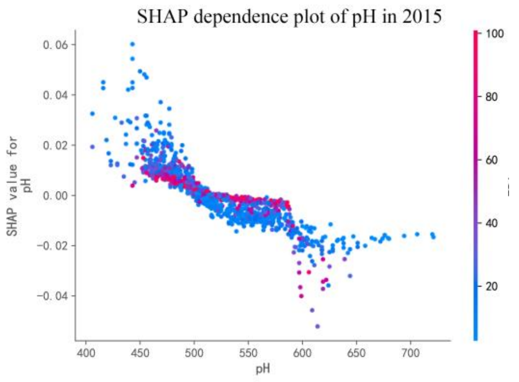


**Figure S5.** SHAP dependence plots of PCY, SOC, and pH across selected time points. Each plot illustrates the relationship between feature values (PCY, SOC, or pH) and their SHAP impact on EH predictions. The x-axis represents the feature value, and the y-axis indicates the corresponding SHAP value. Color gradients represent the values of the most relevant interacting variables, enabling the visualization of potential interaction effects at different time points

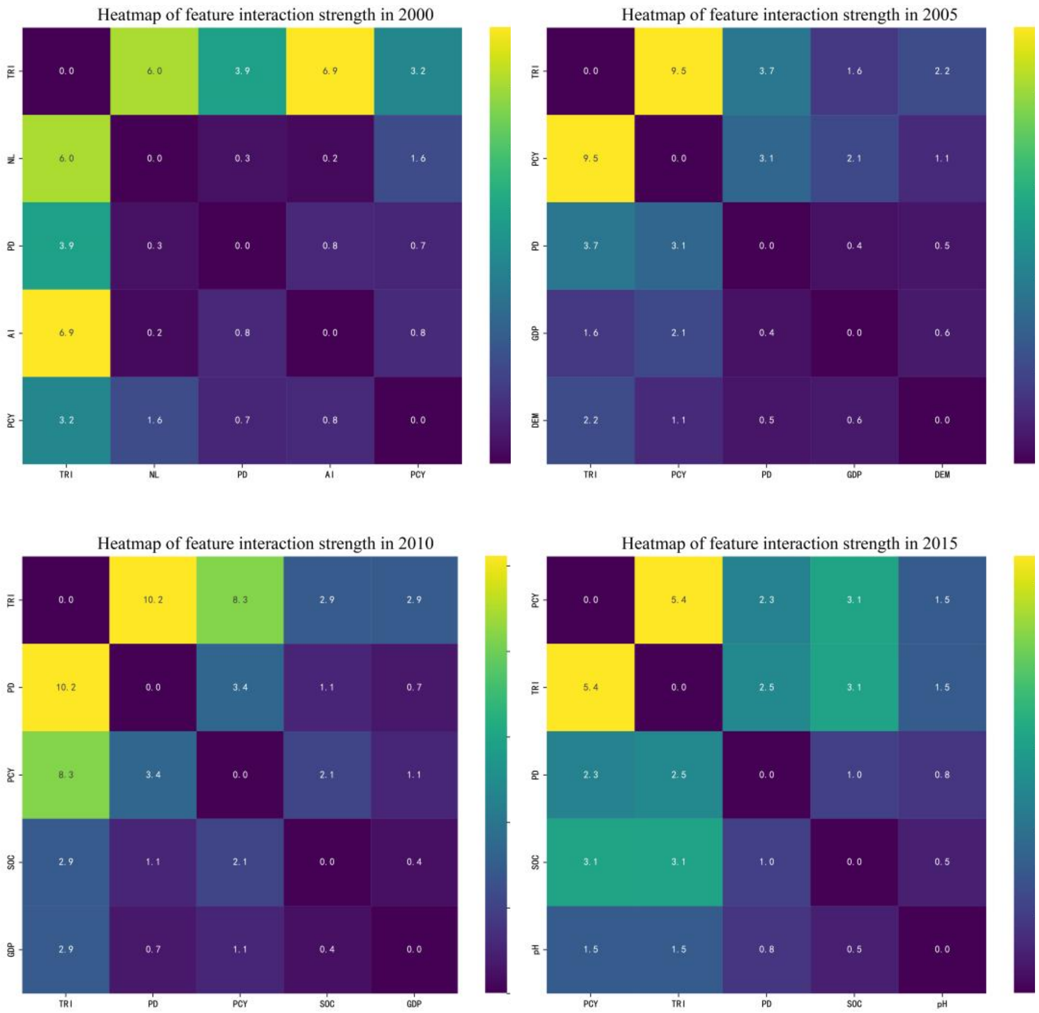

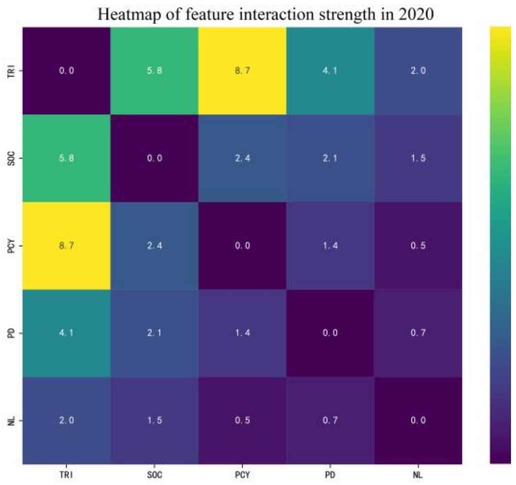


**Figure S6.** SHAP interaction heatmap of driving factors across five time points. Each heatmap shows the magnitude of pairwise interaction effects between variables on EH predictions, revealing dynamic and non-linear relationships over time.

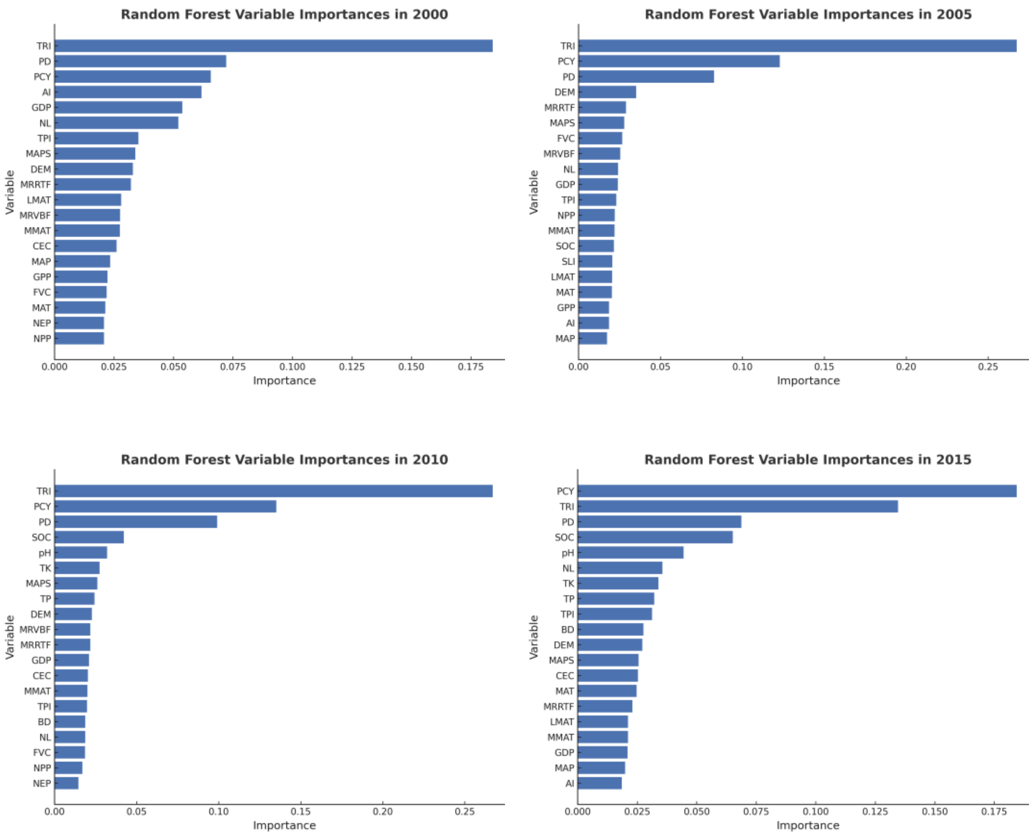

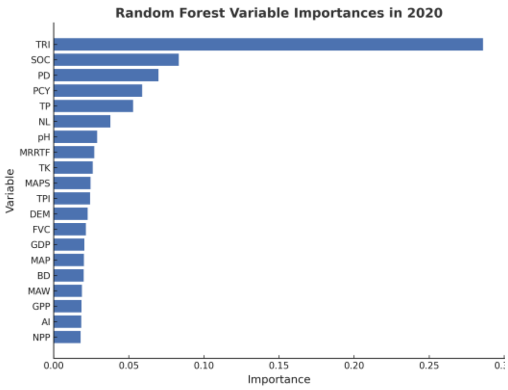


**Figure S7.** Top 20 Random Forest Variable Importances across Five Time Points. The ranking of variable importances based on the Random Forest model is shown for five time points. The top 20 variables are presented, and their relative importance scores are listed. The ranking results show a consistent trend with those derived from SHAP values, indicating robust identification of influential features.
